# Interrogating Low Barrier Hydrogen Bonds with Neutrons, X-rays, and Computation

**DOI:** 10.1063/4.0001112

**Published:** 2025-10-27

**Authors:** Jiusheng Lin, Oksana Gerlits, Daniel W. Kneller, Kevin L. Weiss, Leighton Coates, Mark A. Hix, Solomon Y. Effah, Andrey Kovalevsky, Alice R. Walker, Mark A. Wilson

**Affiliations:** 1 University of Nebraska, Lincoln, NE, Taiwan; 2 Tennessee Wesleyan University, Athens, TN, USA; 3 Oak Ridge National Laboratory, Oak Ridge, TN, USA; 4 Wayne State University, Detroit, MI, USA; 5 University of Nebraska, Lincoln, NE, USA

## Abstract

Low barrier hydrogen bonds (LBHBs) are H-bonds where the barrier to proton transfer from the donor to the acceptor atom is comparable to the quantum mechanical zero-point energy of the interaction, resulting in proton delocalization. Facile proton transfer makes LBHBs effective mechanisms for facilitating acid-base catalysis in certain enzymes, creating favored pathways of allosteric communication between distant sites during catalysis, and imparting a high degree of selectivity in binding certain substrates. How the microenvironment of H-bonded residues affects the degree of proton delocalization in a candidate LBHB is not fully understood. We use two homologous proteins, human DJ-1 and E. coli YajL, as a paired model system to investigate microenvironmental effects on LBHB formation. Neutron diffraction demonstrates that YajL has an Asp- Glu LBHB while the analogous residues in DJ-1 form a conventional H-bond, validating bond length analysis of atomic resolution X-ray crystal structures. We explored the influence of the microenvironment on LBHB character by using large quantum mechanics-molecular mechanics (QM/MM) simulations where the QM region spanned several residues. These simulations support the neutron and X-ray crystallography results, demonstrating that surprisingly distant H-bonding residues can influence the degree of proton delocalization in candidate LBHBs. With the greater accessibility of QM/MM methods, combining simulation and structurally guided sequence analysis may permit the engineering of H-bonds with desired degrees of proton delocalization.

